# The role of microRNAs in regulating neuronal connectivity

**DOI:** 10.3389/fncel.2013.00283

**Published:** 2014-01-03

**Authors:** Hui Chiu, Amel Alqadah, Chieh Chang

**Affiliations:** Division of Developmental Biology, Cincinnati Children’s Hospital Research FoundationCincinnati, OH, USA

**Keywords:** miRNAs, neuronal connectivity, axon pathfinding, axon branching, timing mechanisms, temporal regulation, heterochronic miRNAs, axon outgrowth

## Abstract

The assembly of functional neural circuits is critical for complex thoughts, behavior and general brain function. Precise construction of neural circuits requires orderly transition of sequential events from axon outgrowth, pathfinding, branching, to synaptogenesis. Each of these steps is required to be tightly regulated in order to achieve meticulous formation of neuronal connections. MicroRNAs (miRNAs), which silence gene expression post-transcriptionally via either inhibition of translation or destabilization of messenger RNAs, have emerged as key regulators of neuronal connectivity. The expression of miRNAs in neurons is often temporally and spatially regulated, providing critical timing and local mechanisms that prime neuronal growth cones for dynamic responses to extrinsic cues. Here we summarize recent findings of miRNA regulation of neuronal connectivity in a variety of experimental platforms.

The diverse behaviors of organisms rely on fast information processing performed by the brain. Trillions of neurons comprising the brain form complex networks that enable animals to exhibit consciousness, accumulate memories, engage in learning, and adopt behaviors. The complexity of brain networks is greatly increased by the facts that one neuron can influence its target through multiple pathways (Gutierrez et al., 2013), and that common neurons shared by divergent circuits can modulate reciprocal inhibition between two mutually exclusive behaviors in response to environmental stimuli (Mann et al., 2013). The former case is achieved by the direct and indirect connections between two neurons via two or more synaptic routes and the latter depends on the appropriate information flow from sensory neurons to interneurons as well as from interneurons to motor neurons (Gutierrez et al., 2013; Mann et al., 2013). Both cases indicate the importance of precise connections between neurons to brain function. Precise neuronal connectivity is established through transition of sequential events from axon growth to synapse formation. Axons are attracted to targets, but upon arrival they must switch their responsiveness to guidance cues at the targets such that they are no longer sensitive to these cues, in order to stop outgrowth and form synaptic contacts (Tessier-Lavigne and Goodman, 1996; Stein and Tessier-Lavigne, 2001; Dickson, 2002; Chen et al., 2008). Miswiring of the nervous system can result in serious neurological deficits, such as autism, Parkinson’s, and Alzheimer’s diseases (Hoogland et al., 2003; Lesnick et al., 2007; Zikopoulos and Barbas, 2010). Thus, studying how neurons connect with each other to establish functional circuitry can help us better understand how animal behaviors go awry and may provide insights into potential therapeutic targets for neurological disorders.

## NEURAL CIRCUIT ASSEMBLY

Neurons connect with targets through a series of events: axon initiation, axon pathfinding, axon branching, and synapse formation (Carmeliet and Tessier-Lavigne, 2005; Kolodkin and Tessier-Lavigne, 2011). A single axon grows out from the cell body, and forms a highly motile structure called the growth cone at its tip. The growth cone navigates along the stereotypical pathway via interactions with a variety of guidance cues present in the environment and travels a long distance to reach the target with remarkable precision. Upon reaching the target, the growth cone turns into a presynaptic terminal to form a connection with the target, and the axon starts branching extensively to establish an intricate pattern of connectivity. Each step of neuronal circuit assembly involves local protein synthesis (Campbell and Holt, 2001; Jung et al., 2012). Specific mRNAs are anterogradely transported from the cell body to the axon and to the growth cone to construct local transcriptomes (Zivraj et al., 2010; Gumy et al., 2011; Willis et al., 2011; Cajigas et al., 2012). The expression of mRNAs is tightly controlled by many post-transcriptional regulatory mechanisms, of which microRNA (miRNA)-mediated gene repression is one of them (Bushati and Cohen, 2007; Deglincerti and Jaffrey, 2012; Jung et al., 2012). The fast and dynamic changes in the local proteome enable growth cones to respond rapidly to diverse environmental cues, resulting in elongation, turning, or collapse of growth cones (Campbell and Holt, 2001; Hengst et al., 2009; Andreassi et al., 2010; Zivraj et al., 2010). The post-transcriptional regulators, therefore, play a pivotal role in the establishment of neuronal connections.

## miRNAs AS VERSATILE AND REVERSIBLE REGULATORS OF GENE EXPRESSION IN NEURONS

MiRNA-mediated gene regulation is involved in many aspects of neuronal development and function (Kosik, 2006; Schratt, 2009; McNeill and Van Vactor, 2012). Recent studies have worked out a few molecular mechanics of miRNA-mediated gene silencing (Fabian and Sonenberg, 2012). These small non-coding RNAs bind to the 3′UTR of target mRNAs and repress gene expression by interfering with stability or inhibiting translation of mRNAs (Bartel, 2009). The pleiotropy, speed, and reversibility are features that contribute to the unique regulatory niche of miRNA-based gene regulation in the nervous system (Hobert, 2008). First, individual miRNAs can target multiple genes at the same time to cause broad and significant changes in neuronal transcriptomes (Brennecke et al., 2005; Giraldez et al., 2006). On the other hand, each gene can be targeted by different miRNAs, and/or may contain more than one binding site of the same miRNA, allowing miRNAs to “fine-tune” the level of gene expression (Bartel and Chen, 2004; Hon and Zhang, 2007). Second, the effect of miRNA-mediated gene repression is instant because miRNAs can shut down protein synthesis of the target genes at ribosomes (Pillai et al., 2005). The small size and non-coding nature also allows fast production of miRNAs as compared to transcription factors (Hobert, 2008). Lastly, the miRNA-mediated gene repression can be easily relieved by translocating the targeted mRNA from a miRNA-hijacked ribosome to an active one (Bhattacharyya et al., 2006). In summary, miRNAs provide a local, versatile, fast and reversible mechanism that is sensitive to extracellular stimuli and provides exquisite control of local protein dynamics and axon development.

## SPATIAL AND TEMPORAL ROLES OF miRNAs IN NEURONAL CONNECTIVITY

MiRNA expression is either spatially restricted or temporally regulated in neuronal development. The spatially restricted expression of miRNAs within neurons suggests roles for miRNAs in diverse differentiation events from axon pathfinding to synapse formation. Many miRNAs are expressed specifically in the nervous system, and even in distinct neuronal subsets, implying potentially unique roles in specific cell types. (Lagos-Quintana et al., 2002; He et al., 2012). Furthermore, miRNAs can be localized to subcellular compartments, such as axons, growth cones or synapses, to rapidly alter local gene expression profiles, but not much is known about the transport mechanisms that provide that specificity (Corbin et al., 2009; Natera-Naranjo et al., 2010; Kaplan et al., 2013; Sasaki et al., 2013). The temporally regulated expression of miRNAs within neurons suggests a role in the orderly transition of sequential differentiation events (Johnston and Hobert, 2003; Chang et al., 2004b; Hsieh et al., 2012; Zou et al., 2012, 2013; Chiu and Chang, 2013). Recent evidence indeed showed that miRNAs can provide timing mechanisms for orderly developmental events in neurons (Zou et al., 2012). Significant changes in the expression level of some brain-specific miRNAs during cortical neurogenesis have been reported previously (Krichevsky et al., 2003), suggesting that gene expression profiles may be controlled by the up- or down-regulation of certain miRNAs at each stage of brain development. Thus, the compartmentalized expression of miRNAs provides subcellular control of local gene expression for specific neuronal differentiation events while the temporal constraint of miRNA expression allows for the correct transition timing of sequential differentiation events.

## CONTROL OF AXON OUTGROWTH BY miRNAs

The neural circuit assembly begins with axon outgrowth. In the early phase of neuronal development, a neuron first forms multiple naïve neurites around the soma. One of the neurites will be specified as an axon and extend further while the remaining become dendrites (Craig and Banker, 1994). Actin and microtubule dynamics are required for the neurite formation and elongation (Bradke and Dotti, 1999; Inagaki et al., 2001). Therefore, it is not surprising that miRNAs affect axon initiation or elongation by targeting regulators of the cytoskeleton (**Table 1**). miR-132, for example, induces neurite sprouting of cortical neurons. It does so by inhibiting the p250 GTPase-activating protein that acts upstream of small GTPases Cdc42 and RhoA, to regulate neuronal morphogenesis (Nakazawa et al., 2003; Vo et al., 2005). miRNAs can also regulate axon outgrowth by modulating local protein synthesis. miR-9 locally represses the translation of the microtubule-associated protein 1b (Map1b) in axons to control axon elongation of mouse cortical neurons (Dajas-Bailador et al., 2012). Overexpression of miR-9 decreases the Map1b regulatory effects on axonal microtubules, resulting in the reduction of axon length (Dajas-Bailador et al., 2012; Tymanskyj et al., 2012). miR-19a, a major member of the miR-17-92 cluster, acts in axons to down-regulate the protein level of phosphatase and tension homolog (PTEN) and activate phosphorylated mammalian target of rapamycin (mTOR) pathway (Zhang et al., 2013). mTOR activity is known to be required for local protein synthesis in axonal development and regeneration (Campbell and Holt, 2001; Park et al., 2008). Thus, miR-17-92 cluster promotes axon outgrowth by activating local protein synthesis (Zhang et al., 2013). Although several miRNAs have been shown to affect axon development, few have been shown to play a role specifically in the axon compartment (Aschrafi et al., 2008; Dajas-Bailador et al., 2012; Zhang et al., 2013). In addition, miRNAs also contribute to the temporal regulation of axon outgrowth. In *Caenorhabditis* elegans, the hermaphrodite specific neuron (HSN) projects a single axon to the ventral nerve cord in the fourth larva (L4) stage (Adler et al., 2006). The heterochronic miRNA *lin-4* acts cell-autonomously in HSN neurons to control the timing of axon formation (Olsson-Carter and Slack, 2010). No axon is extended at the L4 stage in *lin-4* mutants while precocious axon outgrowth at the third larval stage occurs in animals over-expressing *lin-4* in HSN neurons (Olsson-Carter and Slack, 2010). The targets of the *lin-4* miRNA, *lin-14* and *lin-28*, inhibit differentiation of HSN neurons. Thus, the *lin-4* miRNA signals axon outgrowth only after HSN neuronal fate is committed (Olsson-Carter and Slack, 2010).

**Table 1:** Summary of miRNA functions in axon development.

MicroRNAs	Functions	Targets	Reference
**Axon outgrowth**
miR-132	Promote axon outgrowth	p250 GTPase-activating protein	Vo etal. (2005)
miR-9	Inhibit axon outgrowth	Microtubule-associated protein 1b (Map1b)	Dajas-Bailador etal. (2012)
miR-17-92 cluster	Promote axon outgrowth	Phosphatase and tensin homolog (PTEN)	Zhang etal. (2013)
*lin-4*	Promote axon outgrowth	LIN-14 and LIN-28 transcription factors	Olsson-Carter and Slack (2010)
**Axon guidance**
*lin-4*	Turn off the growth cone sensitivity to Netrin	LIN-14 transcription factor	Zou etal. (2012)
miR-124	Turn on the growth cone sensitivity to Sema3A	CoREST	Baudet etal. (2012, 2013)
**Axon branching**
miR-9	Promote axon branching	Map1b	Dajas-Bailador etal. (2012)
miR-124	Promote axon branching	Small GTPase RhoG	Franke etal. (2012)

## miRNA REGULATION OF AXON PATHFINDING

Axons are directed to their synaptic targets via the guidance of attractive or repellent cues presented in the environment (Tessier-Lavigne and Goodman, 1996). The sensitivity of axons to guidance cues is determined by the expression of the corresponding receptors in growth cones (Dickson, 2002). To prevent the axon from stalling at intermediate targets or overshooting, the receptor expression needs to be tightly controlled in a timely manner (Zou et al., 2000; Stein and Tessier-Lavigne, 2001). Here we discuss the role of miRNAs in regulating the growth cone responsiveness to two prominent guidance molecules, netrins and semaphorins, during axon pathfinding.

**FIGURE 1:**
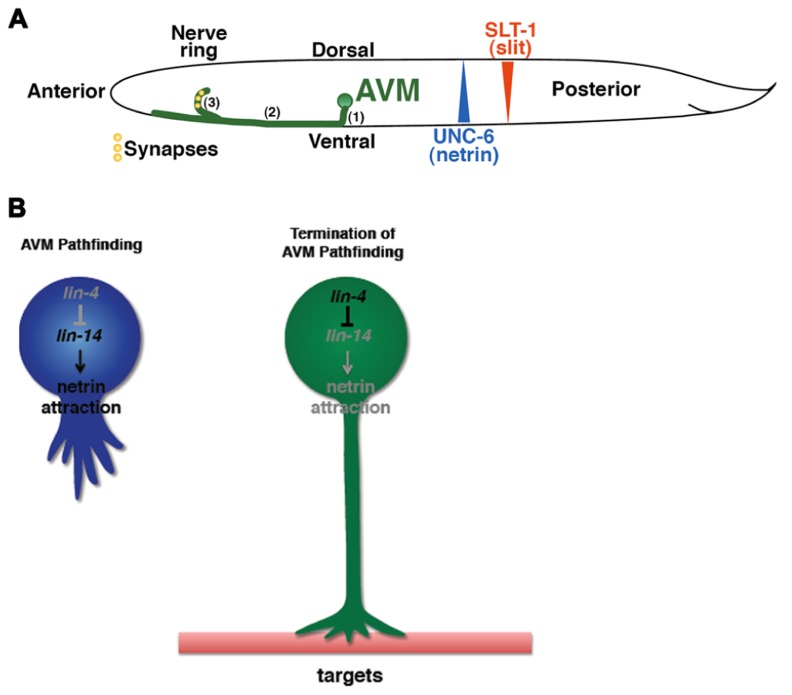
**Identification of the timing mechanism for orderly AVM axon connectivity. (A)** Sequential events of AVM axon outgrowth and synapse formation in the first larval (L1) stage of *C. elegans*. (1) Axon grows ventrally. (2) Axon grows anteriorly. (3) End of axon pathfinding and initiation of synapse formation in the nerve ring. **(B)** The *lin-4–lin-14* regulatory circuit signals the end of axon pathfinding.

Netrins are highly conserved guidance molecules that can function as both attractants and repellents in many stage-dependent biological events, such as axon guidance and motile cell migration (Hedgecock et al., 1990; Ishii et al., 1992; Chan et al., 1996; Kolodkin and Tessier-Lavigne, 2011), but the timing mechanism that controls the responsiveness of growth cones or migrating cells to Netrins at precise times is not fully understood. The axon of the *C. elegans* anterior ventral microtubule (AVM) sensory neurons is guided to the ventral midline through combined actions of Slit repulsion from the dorsal body wall muscles and netrin attraction to the ventral nerve cord (Chang et al., 2004a). Once reaching the ventral midline, the AVM axon projects anteriorly to the nerve ring where it stops outgrowth and forms synapses (**Figure 1A**). An unexpected role was recently reported for the conserved heterochronic miRNA *lin-4* and its target the LIN-14 transcription factor in AVM neuronal connectivity (Zou et al., 2012). Through genetic analysis of a well-characterized AVM axon ventral guidance event and a less characterized AVM synapse formation event, it was shown that *lin-4* functions as a potent and specific negative regulator of netrin signaling in AVM neuronal connectivity by targeting the LIN-14 transcription factor to control the availability of the netrin receptor UNC-40/DCC (Deleted in Colorectal Cancer; **Figure 1B**; Zou et al., 2012). It was well known that heterochronic genes are used in timing mitotic cell development required for molting in worms and embryonic stem cells self-renewal in mice. These results show that these heterochronic genes are re-used in postmitotic neurons to time their differentiation events.

The Semaphorin family contains both secreted and transmembrane proteins that can function in long- or short-range guidance (Yazdani and Terman, 2006; Kolodkin and Tessier-Lavigne, 2011). Many Semaphorins bind the major receptor, Plexins, solely to mediate axonal repulsion. However, some of the secreted Semaphorins, such as Sema3A, bind to the co-receptor Neuropilins, instead (Kolodkin and Tessier-Lavigne, 2011). It has been shown that Sema3A is able to induce growth cone collapse at a specific stage of retinal ganglion cell (RGC) development in *Xenopus* (Campbell et al., 2001). The Sema3A responsiveness depends on the up-regulation of neuropilin-1 (NPR-1) receptor, which is indirectly controlled by the miRNA, miR-124, in a timely manner (Campbell et al., 2001; Baudet et al., 2012, 2013). The Repressor element-1 silencing transcription factor (REST) works with the REST corepressor 1 (CoREST) to inhibit the NPR-1 expression in young RGCs (Baudet et al., 2012, 2013). As the development progresses, the up-regulation of miR-124 accelerates the decay of CoREST mRNA, thereby attenuating the repression of NPR-1 expression (Baudet et al., 2012, 2013). The onset of Sema3A sensitivity of RGC growth cones is delayed in miR-124 knockdown, which is attributed to the retarded expression of NPR-1 (Baudet et al., 2012, 2013). Thus, miR-124 acts as an intrinsic timer to turn on the responsiveness of RGC growth cone to Sema3A by up-regulating NPR-1.

Slit is another key guidance molecule that is able to elicit axonal repulsion from a distance (Kolodkin and Tessier-Lavigne, 2011). The miRNA regulation of Slit signaling has been reported in the context of vascular patterning, but whether miRNAs control Slit signaling at specific times is unknown (Small et al., 2010). Even though no evidence has been shown that the Slit signaling is subject to the miRNA regulation in the context of axon guidance, it is likely that miRNAs may modulate Slit-mediated axon guidance since many molecular mechanisms are shared between vascular and axonal patterning (Carmeliet and Tessier-Lavigne, 2005).

## UNIQUE ROLES OF miRNAs IN AXON BRANCHING

Once axons are in the vicinity of synaptic targets, they slow down outgrowth and branch out to form elaborate neural networks (Acebes and Ferrús, 2000). Similar to axon outgrowth, axon branching relies on cytoskeleton rearrangement, so cytoskeletal regulators likely coordinate both events in response to environmental cues. As mentioned above, miR-9 regulates microtubule dynamics by targeting the Map1b to control axon outgrowth (Dajas-Bailador et al., 2012). The miR-9 level in mouse cortical neurons is regulated by the brain-derived neurotrophic factor (BDNF) that functions as a chemoattractant and also a branching factor (Hoshino et al., 2010; Panagiotaki et al., 2010). In the initial phase of cortical neuron development *in vitro*, shorter exposure to BDNF results in reduced miR-9 expression, which leads to high level of Map1b expression and steady axon outgrowth. Prolonged exposure to BDNF, which could mimic the target recognition phase *in vivo*, results in elevated miR-9 expression, which leads to axon branching as a result of the repressed Map1b expression (Dajas-Bailador et al., 2012). In hippocampal neurons, it was shown that miR-124 regulates axon and dendrite branching by targeting the small GTPase RhoG (Franke et al., 2012). The RhoG activity inhibits axon branching via the ELMO/Dock180/Rac1 pathway, while reducing dendrite branching through the Cdc42 signaling. Therefore, expression of miR-124 in hippocampal neurons promotes axon and dendrite branching (Franke et al., 2012).

## CONCLUDING REMARKS

The exquisite precision with which neural circuits are assembled is crucial for proper brain function, as inappropriate wiring in the nervous system results in various debilitating neurological diseases. Neurons are able to form appropriate connections using processes that involve spatial and temporal regulatory mechanisms, which ensure rapid responses to diverse environmental cues and faithful transition of sequential events in neuronal connectivity. miRNAs have proven to be essential and efficient regulators at several steps of this integral process due to their spatiotemporal specificity, versatile targeting, speed of gene repression, and ease of reversibility. Further study on how miRNAs contribute to the formation of neural circuits will ultimately provide insights into how mis-wiring by miRNA mis-regulation can lead to diseases.

## Conflict of Interest Statement

The authors declare that the research was conducted in the absence of any commercial or financial relationships that could be construed as a potential conflict of interest.
